# ENSO-induced co-variability of Salinity, Plankton Biomass and Coastal Currents in the Northern Gulf of Mexico

**DOI:** 10.1038/s41598-018-36655-y

**Published:** 2019-01-17

**Authors:** Fabian A. Gomez, Sang-Ki Lee, Frank J. Hernandez, Luciano M. Chiaverano, Frank E. Muller-Karger, Yanyun Liu, John T. Lamkin

**Affiliations:** 1 Division of Coastal Sciences, University of Southern Mississippi, Ocean Springs, MS USA; 2 Northern Gulf Institute, Mississippi State University, Stennis Space Center, MS USA; 3 Atlantic Oceanographic and Meteorological Laboratory, NOAA, Miami, FL USA; 4 College of Marine Science, University of South Florida, St Petersburg, FL USA; 5 Climate Prediction Center, NOAA/NWS/NCEP, College Park, MD USA; 6 Innovim, LLC, Greenbelt, MD USA; 7 Southeast Fisheries Science Center, NOAA, Miami, FL USA

## Abstract

The northern Gulf of Mexico (GoM) is a region strongly influenced by river discharges of freshwater and nutrients, which promote a highly productive coastal ecosystem that host commercially valuable marine species. A variety of climate and weather processes could potentially influence the river discharges into the northern GoM. However, their impacts on the coastal ecosystem remain poorly described. By using a regional ocean-biogeochemical model, complemented with satellite and *in situ* observations, here we show that El Niño - Southern Oscillation (ENSO) is a main driver of the interannual variability in salinity and plankton biomass during winter and spring. Composite analysis of salinity and plankton biomass anomalies shows a strong asymmetry between El Niño and La Niña impacts, with much larger amplitude and broader areas affected during El Niño conditions. Further analysis of the model simulation reveals significant coastal circulation anomalies driven by changes in salinity and winds. The coastal circulation anomalies in turn largely determine the spatial extent and distribution of the ENSO-induced plankton biomass variability. These findings highlight that ENSO-induced changes in salinity, plankton biomass, and coastal circulation across the northern GoM are closely interlinked and may significantly impact the abundance and distribution of fish and invertebrates.

## Introduction

The northern Gulf of Mexico (GoM) is a highly productive region strongly influenced by riverine runoff. River plumes bring freshwater, nutrients, sediments, and particulate and dissolved organic matter, significantly impacting the GoM’s physical and biogeochemical properties^1–5^. The Mississippi-Atchafalaya rivers, in particular, with a combined annual mean flow of 21,524 m^3^ s^−1^, and discharge peaks of 30,000 m^3^ s^−1^ or higher during spring, play a key role in the northern GoM ecosystem, delivering large amounts of nutrients for phytoplankton growth^4^, promoting the generation of a bottom hypoxic layer over the Louisiana-Texas shelf during summer^6^, and driving coastal circulation and vertical stratification^7^. River discharge from other river systems, such as Mobile Bay (1,686 m^3^ s^−1^), Apalachicola (704 m^3^ s^−1^), Sabine (405 m^3^ s^−1^), Pearl (303 m^3^ s^−1^), Pascagoula (286 m^3^ s^−1^), Trinity (254 m^3^ s^−1^), Brazos (225 m^3^ s^−1^), and the Choctawhatchee (187 m^3^ s^−1^), although much smaller than the Mississippi-Atchafalaya rivers, also have a considerable effect on primary production and plankton distribution along the northern GoM shelf^1,8–10^.

Spatial patterns of phytoplankton biomass in the northern GoM often co-vary with associated spatial salinity patterns^1,4,11,12^. This association can be explained by enhanced phytoplankton production due to increased riverine nutrient fluxes and salinity-driven vertical stratification that favors the concentration of phytoplankton biomass in the most illuminated and warmest upper layer of the water column^12^. Thus, changes in river discharge into the northern GoM greatly influence plankton production and the survival of upper trophic level species, including commercially important ones, such as Gulf menhaden (*Brevoortia patronus*) and red snapper (*Lutjanus campechanus*)^13–17^. These changes also modulate the spreading of the bottom hypoxic layer over the Louisiana-Texas shelf^6^.

Large-scale climate variability modes, such as the El Niño Southern Oscillation (ENSO), Pacific Decadal Oscillation (PDO), and Atlantic Multidecadal Oscillation (AMO), influence U.S. precipitation patterns and, consequently, impact river runoff into the northern GoM e.g.^18–23^. On an interdecadal time scale, positive PDO and negative AMO phases increase river discharge into the northern GoM, while the opposite occurs during negative PDO and positive AMO phases^14,20,24^. On an interannual time scale, El Niño generally increases river runoff into the GoM, whereas La Niña decreases river runoff^22,25–27^. As a result, ENSO influences salinity in estuaries and marshes, with low and high salinity conditions linked to El Niño and La Niña conditions, respectively e.g.^25,28^. Although river runoff plays an important role as a driver of alongshore circulation in the northern GoM^7,29^, the impact of AMO, PDO, and ENSO induced runoff anomalies on coastal currents has not been documented.

A few studies have investigated the impacts of large-scale climate modes on biotic components in the northern GoM. On an interdecadal time scale, AMO- and PDO-related variability in river runoff and sea surface temperature (SST) appear to be linked to major ecosystem restructuration events^30^. On an interannual time scale, a potential link between positive satellite chlorophyll anomalies and river discharge has been suggested for some El Niño events and the opposite relationship for some La Niña events^31,32^. In the deep GoM, positive chlorophyll anomalies during the El Niño of 1982-83 were linked to increased northerly winds^33^. However, the relationship between ENSO proxies and satellite chlorophyll in the deep GoM remains elusive^34^.

Previous studies, as discussed above, have suggested that ENSO can influence the northern GoM’s salinity and biotic properties. However, a regional characterization of ENSO-induced anomalies has not been fully addressed for the northern GoM. Particularly, the following three key aspects remain unclear: (1) the seasonal modulation of ENSO signal in salinity and plankton biomass; (2) the asymmetry between El Niño and La Niña impacts; and (3) the coastal circulation anomalies and regional redistributions of ocean tracers in response to changes in river runoff and winds. In this study, we attempt to address these questions by using a three-dimensional ocean-biogeochemical model forced with historical atmospheric flux and river runoff data for the period 1979–2014 (see Methods for ocean-biogeochemical model details), along with satellite chlorophyll data and *in situ* zooplankton biomass observations.

To begin, we describe the leading Empirical Orthogonal Function (EOF) mode of salinity, plankton biomass, and chlorophyll anomalies as spatiotemporal patterns of interannual variability, and examine the correlation between these EOF modes and the surface temperature anomaly in the Niño 3.4 region (N34), the latter a well know index for ENSO variability (details in Methods). We then derive the mean anomaly composites for salinity, plankton biomass, coastal currents, and surface winds during El Niño and La Niña conditions, and evaluate the underlying drivers of ENSO-related changes in coastal circulation. Finally, we examine a potential link between El Nino and enhanced plankton biomass in the surface layer of the deep GoM.

## Results

### Main patterns of salinity and plankton biomass

**Figure 1:**
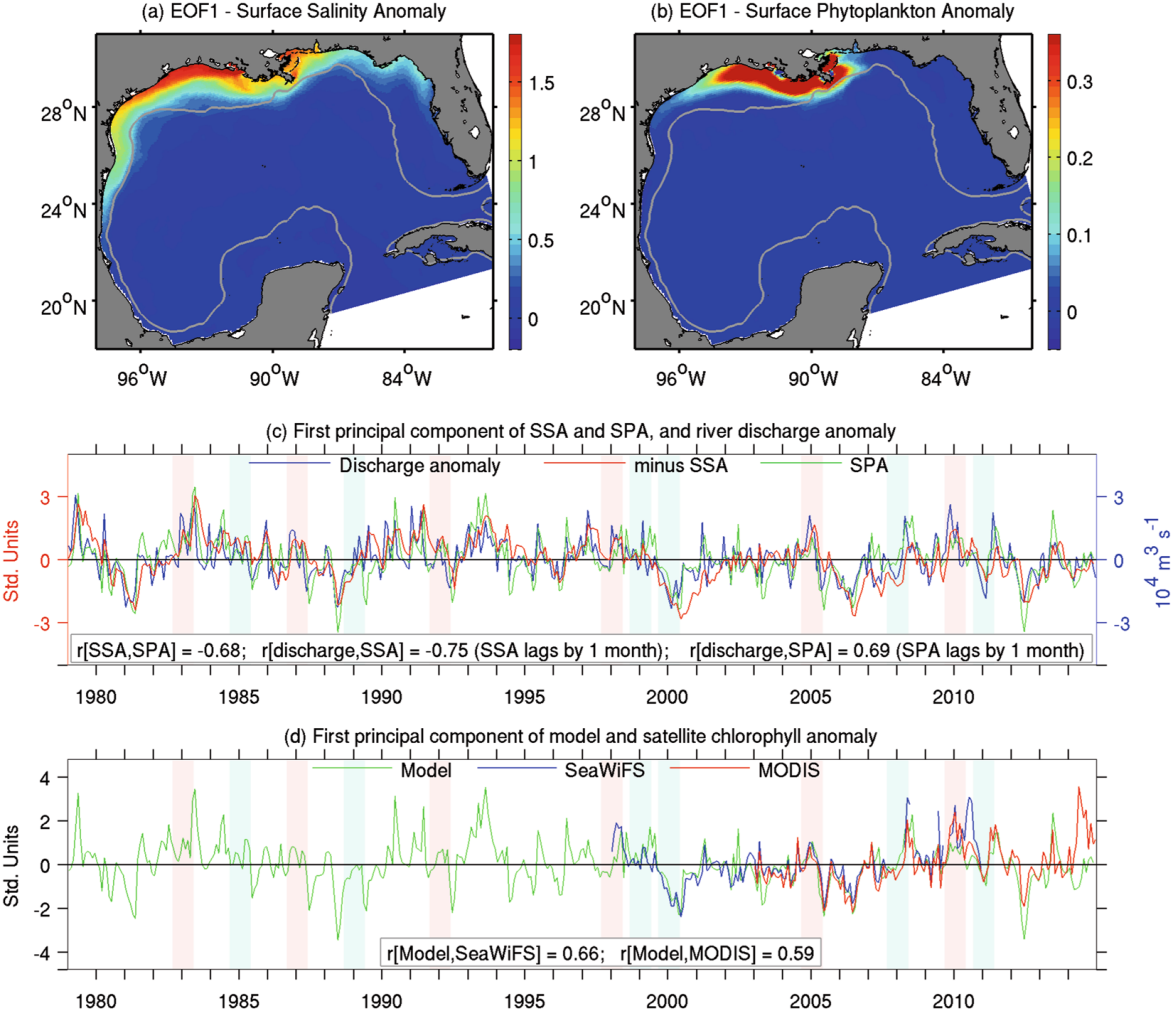
Empirical Orthogonal Function (EOF) patterns of surface salinity anomaly (SSA), surface phytoplankton anomaly (SPA), and chlorophyll anomaly (seasonal cycle removed): (**a**,**b**) First spatial EOF mode of SSA (psu) and SPA (mmol of nitrogen m^−3^). Gray contour depicts the 200-m isobath. (**c**) First principal component series (PC1) of SSA, SPA, and the total river discharge anomaly for northern GoM rivers. (**d**) PC1 of surface chlorophyll derived from model outputs and satellite data (SeaWiFS and MODIS). Fall-to-spring periods that match El Nino and La Nina criteria (see Methods) are highlighted in (**c,d**) as light magenta and cyan shades, respectively. Correlation coefficients (r[x, y]) among time series are indicated in (**c**,**d**).

Figure 1a,b shows the leading EOFs of a surface salinity anomaly (SSA) and a surface phytoplankton anomaly (SPA) (hereinafter anomaly implies data with the climatological annual cycle removed) derived from our ocean-biogeochemical model. These two leading modes are eminently coastal patterns with the largest variability occurring over the Louisiana-Texas inner shelf (Fig. 1a,b). The temporal variation in the EOF mode for these two variables, represented by the Principal Components (PCs), are significantly correlated, making clear the link between salinity and phytoplankton variability over the shelf (Fig. 1c). Both PCs also closely match the variability of the integrated river discharge anomaly from the main northern GoM rivers (Fig. 1c), indicating that the leading driver of interannual variability for salinity and phytoplankton biomass is river runoff. Accordingly, the greatest SSAs and SPAs occur under extreme river discharge conditions during severe drought years (e.g., 1981, 1988, 2000, and 2006) and wet years (e.g., 1979, 1983, and 1991). Positive discharge anomalies, concomitant with negative SSA and positive SPA, prevailed during the 1980s and 1990s relative to the climatology for 1979–2014, indicating an interdecadal modulation of the river runoff signal. The temporal coupling between river discharge and phytoplankton biomass is also observed in the PCs of satellite chlorophyll anomaly derived from the SeaWiFS and MODIS sensors (satellite data description in Methods), which closely resembles the model-derived patterns (Fig. 1d). Similar patterns to those in the model SPA are also found in the model surface zooplankton anomaly (SZA, Supplementary Fig. S1).

### ENSO impacts on the northern GoM

**Figure 2:**
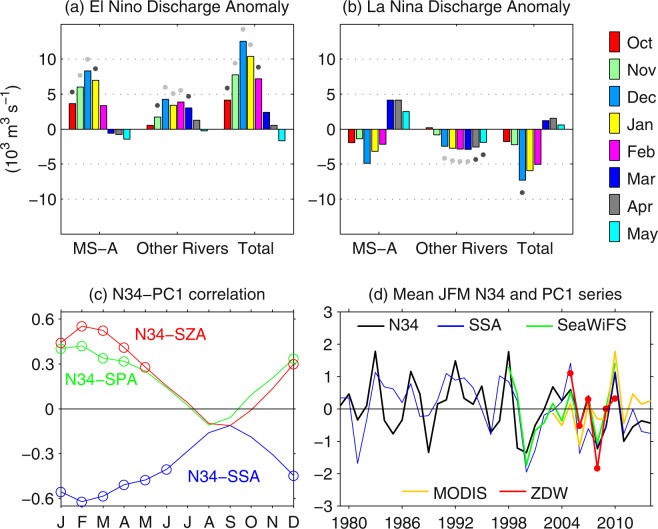
ENSO impact on river runoff, salinity, and plankton biomass: (**a**,**b**) Mean discharge anomalies during El Niño and La Niña for the Mississippi-Atchafalaya rivers (MS-A), rivers other than MS-A (Other Rivers; Table S1), and total rivers (MS-A plus Other Rivers). Dark- and light-gray dots depict the significant correlations at the 90% and 95% confidence levels. (**c**) Monthly variation of the correlation between the El Niño 3.4 SST anomaly (N34) and the PC1 of surface salinity anomaly (SSA), surface phytoplankton anomaly (SPA), and surface zooplankton anomaly (SZA); circles depict significant correlations at the 95% confidence level. (**d**) Mean January-March (JFM) N34 index and the principal component of SSA, the chlorophyll anomaly from SeaWiFS and MODIS, and a standardized time series of zooplankton dry weight (ZDW) for March. The mean and standard deviation of the original (non-standardized) zooplankton dry weight series is 49 and 20 mg m^−3^, respectively.

The influence of ENSO on precipitation patterns over the southeastern continental United States is usually phase-locked to the seasonal cycle, such that the strongest anomalies occur during winter (positive during El Niño and negative during La Niña) e.g.^35–38^. As a consequence, El Niño’s impact on river discharge has a marked seasonality (Fig. 2a), with the largest positive anomalies occurring in late fall and winter and declining values occurring in spring. The sign of the river discharge anomalies reverses during La Niña (Fig. 2b), although La Niña anomalies for the Mississippi-Atchafalaya rivers are non-significant. Since interannual changes in salinity and plankton biomass along the coastal areas of the northern GoM are mainly driven by river discharge (Fig. 1), it is logical to hypothesize that an ENSO signal for salinity and plankton biomass can be expected during winter. To evaluate this hypothesis, the correlation coefficients between the N34 and PC series of the model SSA, SPA, and SZA were estimated for each calendar month. We presented the correlation at zero-lag, but similar results are derived when N34 leads the PC series by 1–4 months (not shown). Consistent with the ENSO signal in river discharge, the correlation patterns between the N34 and PC1 series show a strong seasonal modulation (Fig. 2c), with the maximum correlation in February (*r* = −0.62, 0.48, and 0.58 for SSA, SPA, and SZA, respectively) and statistically significant values occurring only during December-May. The derived patterns are supported by observational data, which also show a significant correlation between the N34 and PC series for SeaWiFS and MODIS chlorophyll (*r* = 0.78 and 0.55 for the January-March averaged time series of SeaWiFs and MODIS, respectively), as well as between the N34 and the *in situ* zooplankton dry weight series from Dauphin Island (*r* = 0.83 for March; see *in situ* zooplankton data in Methods) (Fig. 2d).

**Figure 3:**
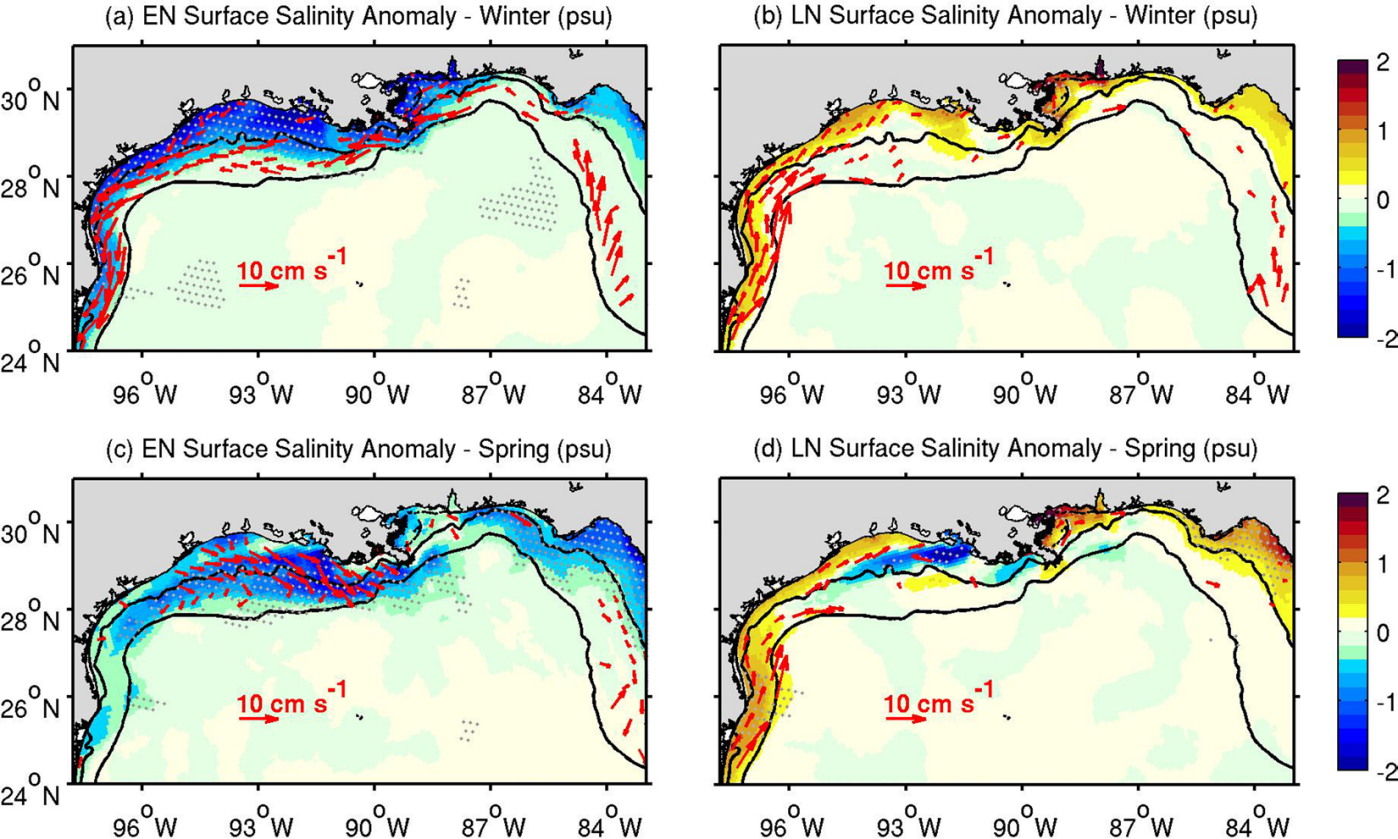
Mean El Niño (EN; **a**,**c**) and La Niña (LN; **b**,**d**) composites for the surface salinity anomaly (SSA, color) and surface shelf current anomaly (red arrows; significant values only) during winter (December–February; **a**,**b**) and spring (March–May; **c**,**d**). Gray dots indicate significant salinity anomalies at the 90% confidence level. Black contours depict the 25- and 200-m isobaths.

To visualize the spatial variability of salinity and coastal circulation due to ENSO, we derived El Niño and La Niña composites of SSA and surface velocity for winter (December-February) and spring (March-May). During El Niño winters (Fig. 3a), the SSA displays significant negative values across most of the northern GoM. The largest anomaly magnitude (about 2 psu) is located along the inner shelf (onshore of the 25-m isobath) off Mississippi, Louisiana, and Texas (87°–96°W). Concurrent with this pattern in salinity, anticlockwise circulation anomalies are observed along the outer shelf (offshore of the 25-m isobath), as well as along the Texas inner shelf. This implies a strengthening of the prevailing westward flow during El Niño on the Louisiana-Texas shelf (the average climatological circulation is shown in Supplementary Fig. S2). The negative winter SSA condition persists throughout spring, but the magnitude of the anomalies decreases significantly nearshore (Fig. 3c). An offshore spread of the salinity anomalies is evident, linked to predominantly southeastward current anomalies. On the other hand, the derived La Niña SSA composite is non-significant across most of the northern GoM shelf (Fig. 3b,d), reflecting the asymmetry between El Niño and La Niña discharge patterns. An examination of the PC1 of the SSA reveals that the weaker La Niña signal is partly explained by the two weak La Niña events in 1984–85 and 1998–99, as fresher conditions prevailed during these events (Supplementary Fig. S3). Still, La Niña composites display the opposite pattern to El Niño composites during winter, but with about half of the El Niño anomaly magnitude. The circulation anomalies linked to La Niña winters are mainly clockwise and located in the northwestern GoM. The saltier pattern breaks in spring, as negative SSAs associated with the Mississippi-Atchafalaya plumes spreads over the Louisiana-Texas shelf (the mean La Niña discharge anomalies for the Mississippi-Atchafalaya rivers are positive during March-May; Fig. 2b). However, positive SSAs are observed nearshore across most of the northern GoM, with the largest values located northeast of the Mississippi delta (~89°W), in the northeastern GoM (83°–85°W), and near the U.S.-Mexico border (~26°N, ~97°W).

**Figure 4:**
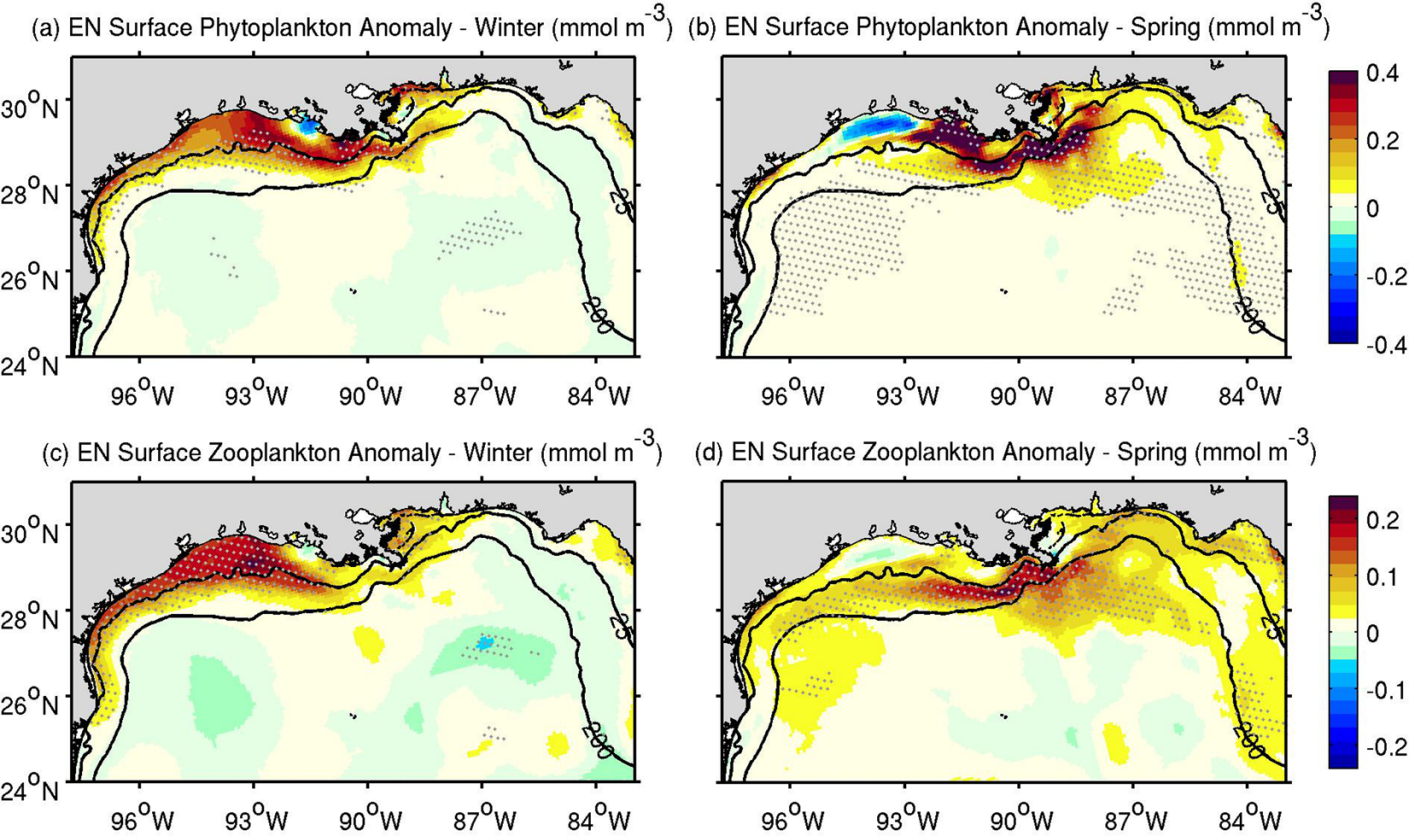
Mean El Niño (EN) composites for the surface phytoplankton anomaly (SPA, **a**,**b**) and surface zooplankton anomaly (SZA, **c**,**d**) during winter (December–February; **a**,**c**) and spring (March–May; **b**,**d**). Phytoplankton concentration is in terms of mmol of nitrogen m^−3^. Gray dots indicate significant anomalies at the 90% confidence level. Black contours depict the 25- and 200-m isobaths.

We also examined spatiotemporal patterns in plankton anomalies induced by ENSO. Circulation patterns significantly influence the distribution of SPA and SZA during El Niño, generating distinct winter and spring patterns (Fig. 4). The enhanced westward advection of the Mississippi-Atchafalaya rivers and other river plumes during El Niño winters determine the largest SPAs and SZAs shoreward of the 25-m isobath and west of 88°W (Fig. 4a,c). On the other hand, southeasterly current anomalies during El Niño springs lead to an increased offshore export of plankton biomass, especially in the north-central GoM (Fig. 4b,d). Because zooplankton growth responds to phytoplankton growth, the largest accumulation rates of zooplankton biomass occur downstream of the phytoplankton biomass maximum, producing the greatest zooplankton anomalies westward from the phytoplankton maximum in winter, and southward in spring. La Niña composites for the SPA show mostly non-significant anomalies across the northern shelf (Supplementary Fig. S4). Consistent with the pattern in salinity, the SPA and SZA during La Niña winters are predominantly negative. This low biomass pattern largely vanishes during La Niña spring, as positive SPAs and SZAs appear over the north-central GoM.

### Drivers of ENSO circulation anomalies

**Figure 5:**
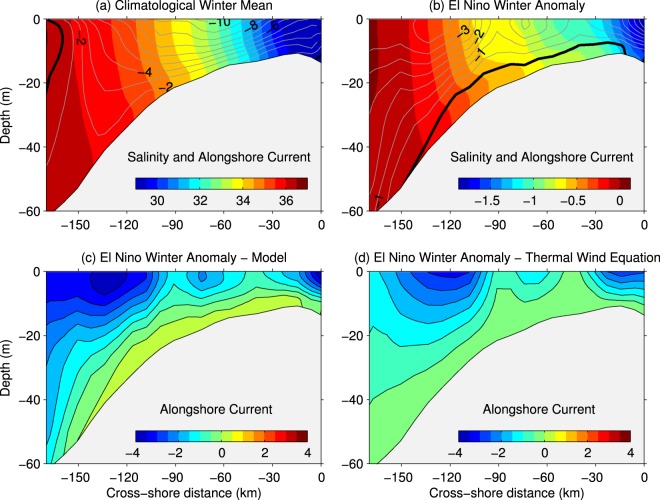
Winter (December–February) vertical patterns for the cross-shore section A on the Louisiana-Texas shelf: (**a**) Model climatological mean and (**b**) El Niño anomaly for salinity (color) and alongshore current (contours; cm s^−1^). (**c**) El Niño alongshore current anomaly. (**d**) El Niño alongshore current anomaly derived from the simulated density field using the thermal wind equation (assuming zero velocity at the bottom). Location of section A is shown in Fig. S5.

On a seasonal time scale, the predominant downwelling favorable winds during winter compress the Mississippi and other river plumes against the coast, inducing a sharp salinity gradient that drives westward flow along the northern GoM^29^. This gradient can be seen in the simulated climatological pattern of salinity and alongshore flow (Fig. 5a) from a vertical section across the Louisiana-Texas shelf (section A, location depicted in Supplementary Fig. S5). There, salinity displays almost vertically-oriented isohalines, ranging from ~28 psu nearshore to >36 psu over the outer shelf (bottom depth >150 m), and the maximum alongshore currents (~10 cm s^−1^ at the surface) occur in response to the strongest salinity gradient. Since the winter alongshore-flow in the northern GoM shelf is, to a great degree, in geostrophic balance^7^, we can hypothesize that the decrease in nearshore salinity and, consequently, the increase in the cross-shore density gradient, drives the westward current increase during El Niño (Figs 3a and 5b,c). To evaluate this hypothesis, we derived geostrophic currents from the thermal wind relationship (see equation (1) in Methods) using the model density field (Fig. 5d). The comparison revealed a similar structure and amplitude of the anomalies for the modeled current and the current derived from the thermal wind balance, with maximum values (~4 cm s^−1^) located nearshore and over the outer shelf (~125 km offshore). This result suggests a strong link between the Louisiana-Texas circulation anomalies and the salinity-driven changes in density during El Niño winters. Similar patterns but with opposite sign (eastward anomalies) and smaller maximum amplitude (~2.5 cm s^−1^) were obtained for La Niña winters (Supplementary Fig. S6).

**Figure 6:**
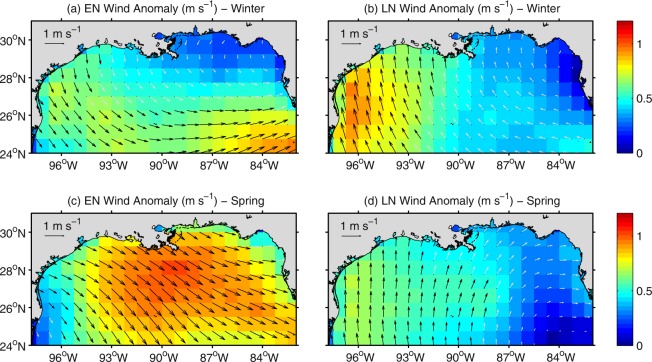
Mean El Niño (EN; **a**,**c**) and La Niña (LN; **b**,**d**) composites for the wind velocity (vectors) and wind speed anomaly (color) at the surface derived from the ERA-interim reanalysis product for winter (December–February, **a**,**b**) and spring (March–May, **c**,**d**). Dark (light) gray arrows depict significant (non-significant) values at the 90% level.

Across the northwestern shelf (southern Texas and northern Mexico coasts), the winter alongshore-flow variability associated with changes in salinity is reinforced by winds. Northerly winds anomalies during El Nino (Fig. 6a) induce onshore Ekman transport, which increases the zonal gradient of sea surface height, triggering an anomalous southward barotropic flow. On the other hand, southerly wind anomalies during La Nina (Fig. 6b) induce offshore Ekman transport and trigger an anomalous northward barotropic flow. The wind influence on circulation can be seen in the velocity patterns of a cross-shore section off southern Texas (section B, location shown in Supplementary Fig. S5), where the thermal wind approximation captures main features in the model flow anomaly but underestimates the anomaly’s magnitude, especially during La Niña (Supplementary Fig. S7). During El Niño spring, the alongshore-current anomalies over the Louisiana-Texas shelf depart from the thermal wind-derived flow anomalies (not shown), and wind-driven barotropic dynamics become more prominent. This is explained by the strengthening of El Niño wind anomalies, which progress from northerly during winter to northwesterly (i.e., upwelling favorable) during spring (Fig. 6a,c), inducing an anomalous southeastward flow into the north-central shelf during spring (Fig. 3c).

### ENSO impacts on the deep GoM

Additional ENSO-related anomalies in plankton biomass can be expected in the surface layers of the deep GoM (bottom depth >500 m), where river inputs are not dominant. Changes in plankton production in the deep GoM are mainly linked to mixing and stratification changes, the latter mostly driven by temperature^12,34^. The link between thermal stratification and phytoplankton biomass is evident in the northern deep GoM series of SSTs, the vertical mixing of nitrate, and surface phytoplankton (Supplementary Fig. S8a; northern deep GoM series are extracted from the deep ocean region north of 25°N), which show positive phytoplankton anomalies associated with cold and increased vertical mixing periods. It is well know that El Niño increases the frequency of cold fronts, determining the northwesterly anomalies shown in Fig. 6, promoting increased vertical mixing and negative temperature anomalies during late winter and early spring^39^ and, consequently, impacting plankton biomass. Indeed, we found significant correlations between N34 and the model derived time series of the vertical mixing of nitrate, SSTs, phytoplankton, and zooplankton anomalies (N34 leading by 3 months) during spring (Supplementary Fig. S8b). This result is consistent with the expected ENSO modulation of plankton biomass due to changes in vertical mixing, as suggested by Melo-Gonzalez *et al*.^33^. This ocean signal reinforces the positive phytoplankton anomalies during El Niño, especially over the outer shelf.

## Summary and Discussion

Using the outputs of a regional high-resolution ocean-biogeochemical model, we determined that the leading mode of salinity and plankton biomass variability in the northern GoM is associated with river discharge variability. The variability in the PC1 time series compares well with the patterns derived from satellite chlorophyll, as well as *in situ* zooplankton biomass observations. We found significant correlations between the EOF modes of surface salinity and plankton biomasses and the Nino3.4 time series. The correlations are largest during winter and early spring, reflecting the seasonal phase locking of ENSO signal. Further composite analysis revealed an asymmetry between El Niño and La Niña impacts. The El Niño-induced anomalies can be more than two times larger than the La Niña-induced anomalies.

Our study reports ENSO-induced anomalies in the coastal circulation over the northern GoM, which has not been address in previous studies. ENSO disturbances in the cross-shore salinity gradient modulate the intensity of the alongshore current in the Louisiana-Texas shelf during winter via thermal wind relationship. ENSO-induce wind anomalies during winter reinforce the alongshore-current anomalies over southern Texas and the northeastern Mexican coast. During El Niño springs, the wind impact on alongshore circulation anomalies is more prominent, and the alongshore-current anomalies over the Louisiana-Texas shelf deviate from the thermal wind relation approximation. These coastal circulation anomalies during El Niño explain the largest plankton anomalies west of 89°W during winter and off the north-central shelf during spring. We also found that ENSO wind anomalies impact the seasonal patterns of mixing and stratification in the deep GoM, and thus modulate plankton biomass during late winter and early spring, consistent with the hypothesis of Melo-Gonzalez *et al*.^33^.

The above-described anomalies in salinity and plankton biomass could have significant impact on the reproductive success and biological condition of upper trophic levels, including commercially important species. Indeed, an improved Gulf menhaden condition (measured as fish oil content) is associated with El Niño years, presumably due to increased prey biomass^17^. Additionally, ENSO disturbances in river discharge and coastal circulation patterns influence the dispersal and recruitment of Gulf menhaden, as previous studies have indicated low recruitment levels associated with increased Mississippi-Atchafalaya river discharge^15,40^. Salinity anomalies may also have a direct impact on fish growth and condition, such as for red snapper larvae that have experienced declining conditions during low salinity periods^16^. Although the link between ENSO and upper trophic level variability has been suggested for several species of fish and invertebrates, the ENSO-related patterns of salinity, plankton biomass, and circulation—three variables hypothesized as driving mechanisms of recruitment and condition variability—have been scarcely described. In this context, our model results provide a framework to better comprehend ENSO-related variability in the northern GoM ecosystem and advance understanding of the larger-scale climate variability mode as a driver of ecosystem and marine population changes.

Finally, ENSO-induced anomalies in river discharge, phytoplankton biomass, and winds could potentially influence hypoxia development over the Louisiana-Texas shelf^41,42^. However, estimations of midsummer hypoxia size during 1985–2011^6^ do not support an evident link between ENSO conditions and hypoxia (not shown). This could be explained by the difference in seasonality between ENSO and hypoxia. More specifically, the strongest ENSO anomalies in river discharge, salinity and plankton biomass occur during winter and early spring, while conditions for the development of bottom hypoxia appear to occur mainly during late spring and early summer^41,43^.

## Methods

### Ocean-Biogeochemical Model

The Regional Ocean Model System^44^ was used to simulate the physical and biogeochemical processes in the northern GoM for 1979–2014. The model domain encompasses the entire GoM and has about 8 km horizontal resolution and 37 sigma-coordinate levels. A third order upstream scheme and a fourth order Akima scheme were used for horizontal and vertical momentum, respectively. A multidimensional positive definitive advection transport algorithm (MPDATA) was used for tracer advection. Vertical turbulence was resolved by the Mellor and Yamada 2.5-level closure scheme. We derived the initial and open boundary conditions from a 25 km horizontal resolution model for the North Atlantic^45^. Surface fluxes of momentum, heat, and freshwater were derived from the European Center for Medium Range Weather Forecast reanalysis product ERA-Interim^46^ using bulk parameterization. River runoff and nutrient loading from 54 river sources in the GoM were explicitly represented. Further model simulation details and validation can be found in Gomez *et al*.^47^.

### Observations

Monthly mean composites of satellite chlorophyll from the NASA Sea-Viewing Wide Field-of-View Sensor (SeaWIFS) and Moderate Resolution Imaging Spectroradiometer (MODIS) were retrieved from the Institute for Marine and Remote Sensing, University of Florida, and processed using standard NASA algorithms (http://imars.usf.edu). All products followed the latest implementation of the atmospheric correction, based on Ding and Gordon^48^. Chlorophyll-a concentration from SeaWiFS and MODIS was estimated using the NASA OC4 and OC3 band ratio algorithms^49^. Monthly observations of zooplankton dry weight were derived from day-time oblique net sampling observations (0.202 mm mesh net) at a location about 20 km south of Dauphin Island, Alabama (see Supplementary Fig. S9). Details on zooplankton sampling are in Carassou *et al*.^50^, and dry weight estimation protocols are in Postel *et al*.^51^. The 3-month running mean time series of the SST anomaly in the Niño 3.4 region (N34) was obtained from the NOAA Climate Prediction center (www.cpc.ncep.noaa.gov). River discharge data from northern GoM rivers were retrieved from the US Geological Survey (USGS; https://waterdata.usgs.gov).

### Statistical analysis

We performed Empirical Orthogonal Function (EOF) decomposition^52^ to extract the main mode of interannual variability in surface anomalies of salinity, plankton biomass, and chlorophyll. EOF analysis is a widely used technique in climate and ocean sciences that uses orthogonal basis functions to describe dominant spatiotemporal modes of variability. Each EOF mode is represented by a spatial pattern (the EOF) and its temporal variability (the Principal Component time series). The leading EOF modes of simulated SSA, SPA, and SZA account for 34%, 31% and 18% of the total variance, respectively. The leading EOF modes of surface chlorophyll anomalies in the model, SeaWiFS, and MODIS explain 35%, 21%, and 20% of the variance, respectively.

To describe the ENSO-related variability in salinity, plankton biomass, ocean currents, and surface winds we estimated mean composite for El Niño and La Niña conditions. The definition of the El Niño/La Niña periods was based on the N34 time series, with warm (cold) ENSO conditions linked to N34 values > 0.5 °C (<−0.5 °C). We only considered El Niño and La Niña events that persisted until late spring (May) because ENSO events that persist throughout the spring have a more significant effect on the atmospheric circulation anomalies that influence U.S. rainfall e.g.^35,53–55^. This criterion was met for six El Niño events (1982-83, 1986-87, 1991-92, 1997-98, 2004-05, and 2009-10) and six La Niña events (1984-85, 1988-89, 1998-99, 1999-00, 2007-08, and 2010-11). The statistical significance of these ENSO events was assessed with Monte Carlo experiments^52^. For each variable, 1,000 independent realizations of the composite were generated by randomly selecting 6 years (six is the number of El Niño/La Niña events) from 1979–2014. An El Niño (or La Niña) composite value was significant at the 90% level when it fell outside the interval defined by the percentiles of 5% and 95% from the randomly generated composite distribution.

### Thermal wind balance

To analyze ENSO-related alongshore flow variability, mean values of the geostrophic current were derived from the model density field using the thermal wind equation:

1 $${u}_{g}(z)=(\frac{g}{{\rho }_{0}\,f}){\int }_{-H}^{z}\frac{\delta \rho }{\delta y}dz$$

where *u*_*g*_(*z*) is the geostrophic current at depth z, *g* is the gravitational acceleration (9.8 m s^−2^), ρ_o_ is a reference density (1,025 kg m^−3^), *f* is the Coriolis parameter, $\frac{\delta \rho }{\delta y}$ is the cross-shore density gradient, and *H* is the bottom depth. Following Zhang *et al*.^7^, it was assumed that horizontal geostrophic velocity vanishes at the bottom.

## Electronic supplementary material

Supplementary Material

## Data Availability

The ocean–biogeochemical model outputs used in this study are in the Network Common Data Form (NetCDF) format on the NOAA-AOML server, available upon request to the corresponding author.
