# Supplementary material for: ENSO-induced co-variability of Salinity, Plankton Biomass and Coastal Currents in the Northern Gulf of Mexico
Source: Sci Rep. 2019 Jan 17;9:178. doi: 10.1038/s41598-018-36655-y (PMC6336811; doi:10.1038/s41598-018-36655-y)
Supplement: Supplementary file 1 — Supplementary Material [file 41598_2018_36655_MOESM1_ESM.pdf]

Supplementary Material for:

ENSO-induced co-variability of Salinity, Plankton Biomass and Coastal Currents in the Northern Gulf of Mexico

Fabian A. Gomez[1,2,3*], Sang-Ki Lee[3], Frank J. Hernandez Jr.[1], Luciano M. Chiaverano[1], Frank E. Muller-Karger[4], Yanyun Liu[5,6], and John T. Lamkin[7]

[1] Division of Coastal Sciences, University of Southern Mississippi, Ocean Springs, MS, USA

[2] Northern Gulf Institute, Mississippi State University, Stennis Space Center, MS, USA

[3] Atlantic Oceanographic and Meteorological Laboratory, NOAA, Miami, FL, USA

[4] College of Marine Science, University of South Florida, St Petersburg, FL, USA

[5] Climate Prediction Center, NOAA/NWS/NCEP, College Park, MD, USA

[6] Innovim, LLC, Greenbelt, MD, USA

[7] Southeast Fisheries Science Center, NOAA, Miami, FL, USA

e-mail: Fabian Gomez (fabian.gomez@noaa.gov)

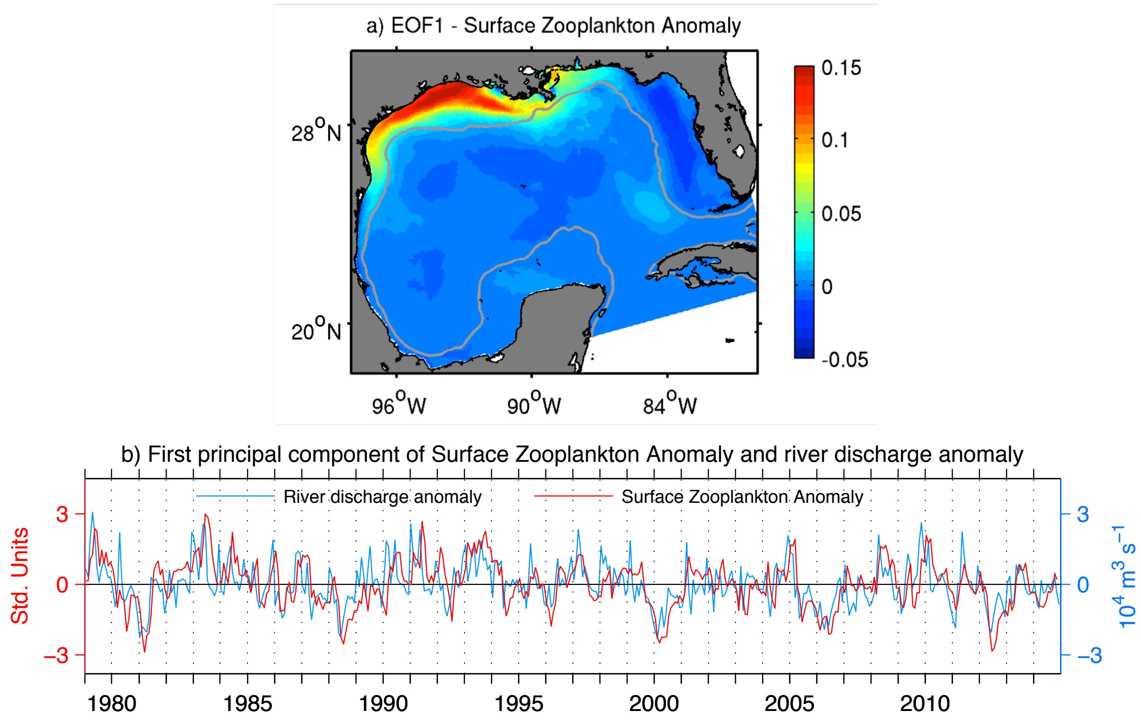

Figure S1. Empirical Orthogonal Function (EOF) patterns of the surface zooplankton anomaly (SZA) model (seasonal cycle removed): a) First spatial EOF mode of the SZA (mmol of nitrogen m^{-3}). Gray contour depicts the 200-m isobath; and b) First principal component series of the SZA and discharge anomaly from northern GoM rivers.

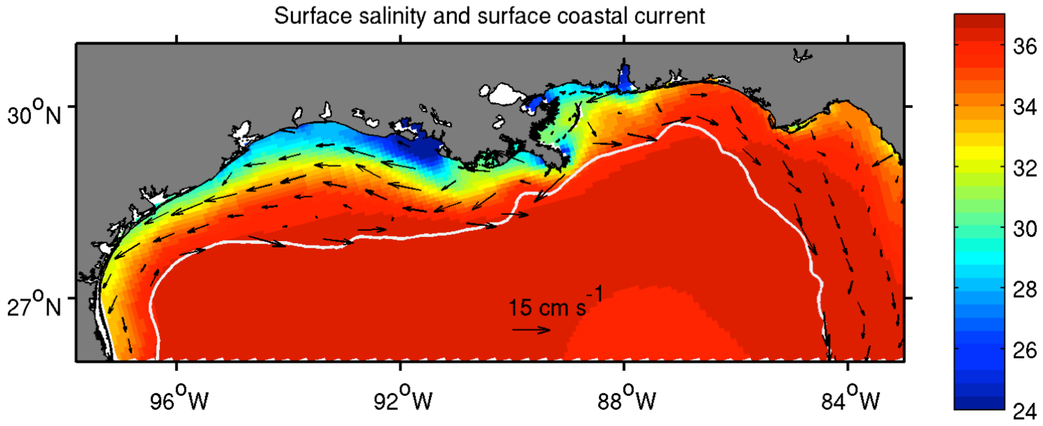

Figure S2. Salinity (color) and surface currents over the shelf (vectors) during January-March. White contour depicts the 200-m isobath.

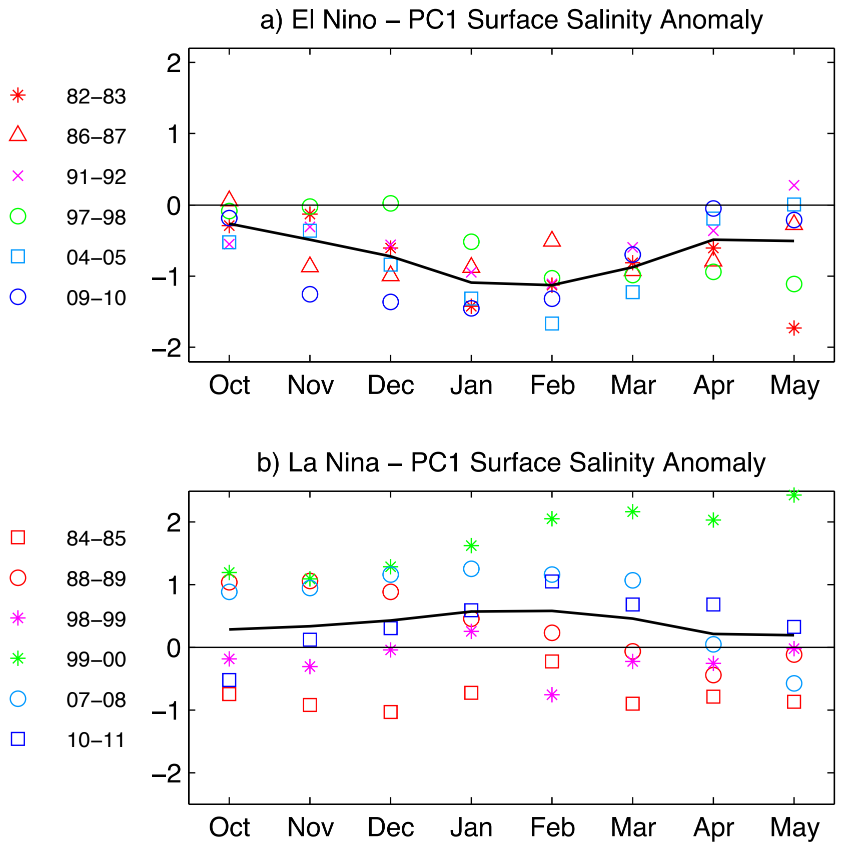

Figure S3. PC1 evolution for the surface salinity anomaly (SSA) during a) El Niño and b) La Niña periods. Only El Niño/La Niña events that prevailed until late spring were included. Black line depicts the monthly EN/LN mean conditions.

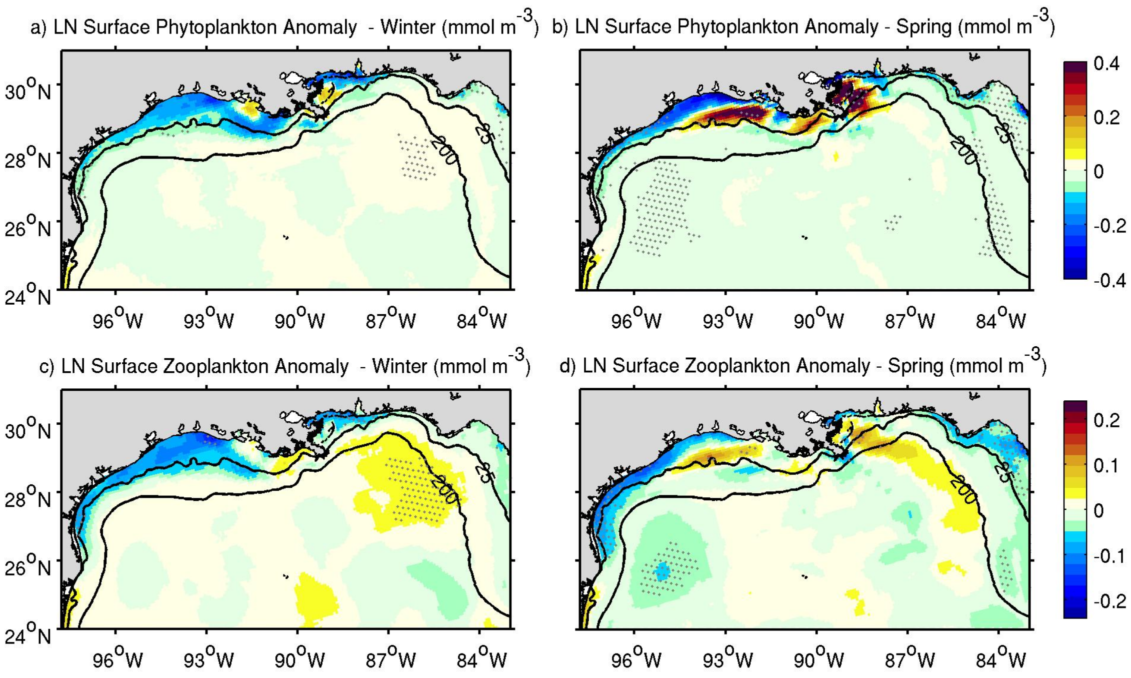

Figure S4. Mean La Niña (LN) composites for the surface phytoplankton anomaly (SPA, a,b) and surface zooplankton anomaly (SZA, c,d) during winter (December-February; a,c) and spring (March-May; b,d). Plankton concentration is in terms of mmol of nitrogen m^{-3}. Gray dots indicate significant anomalies at the 90% confidence level. Black contours depict the 25- and 200-m isobaths.

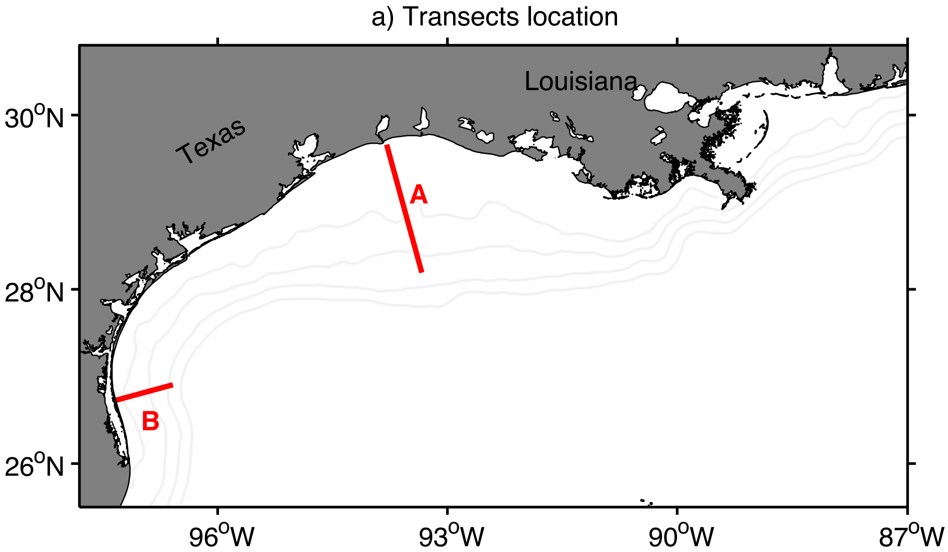

Figure S5. Horizontal location of sections A and B. Gray contours show the 25-, 50-, 100-, and 200-m isobaths.

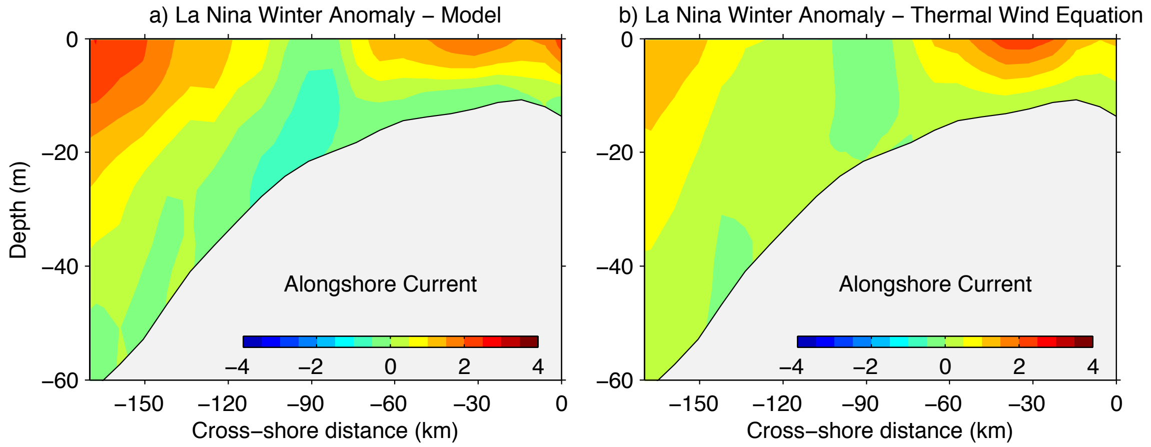

Figure S6. Alongshore current anomaly during La Nina winters (December-February) derived from the a) model and b) thermal wind equation in section A. Horizontal location of section A is shown in Figure S5.

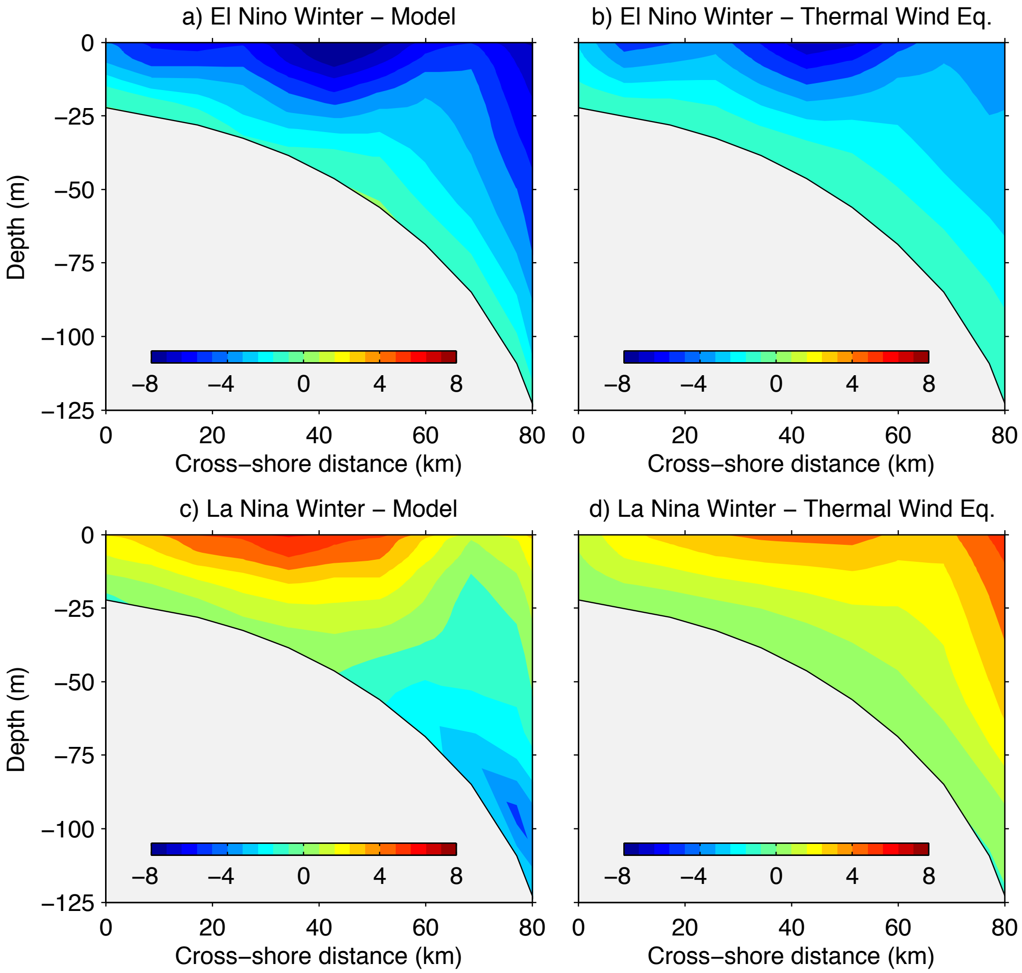

Figure S7. Alongshore current anomaly during El Niño winter (December-February) derived from the a) model and b) thermal wind equation in section B; c-d) as with a-b but for La Niña winter. Horizontal location of section B is shown in Figure S5.

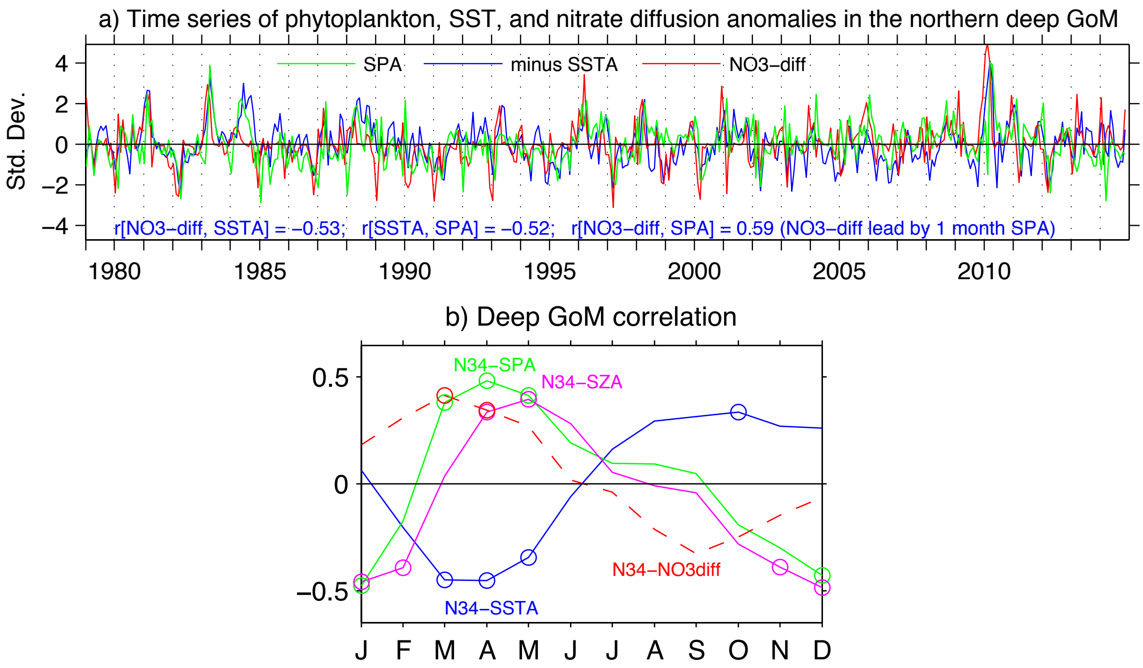

Figure S8. Deep Gulf of Mexico patterns: a) standardized anomalies of surface phytoplankton, SST, and the vertical diffusion of nitrate (SPA, SSTA, and NO3-diff, respectively) from the northern part of the deep GoM (offshore of the 500-m isobath and north of 25°N). NO3-diff is the average within the upper 0-30 m layer. Correlation coefficients (r[x, y]) among the time series are indicated in blue. b) Correlation of N34 with NO3-diff, SSTA, SPA, and SZA for each calendar month. Circles depict significant correlations at the 95% confidence level.

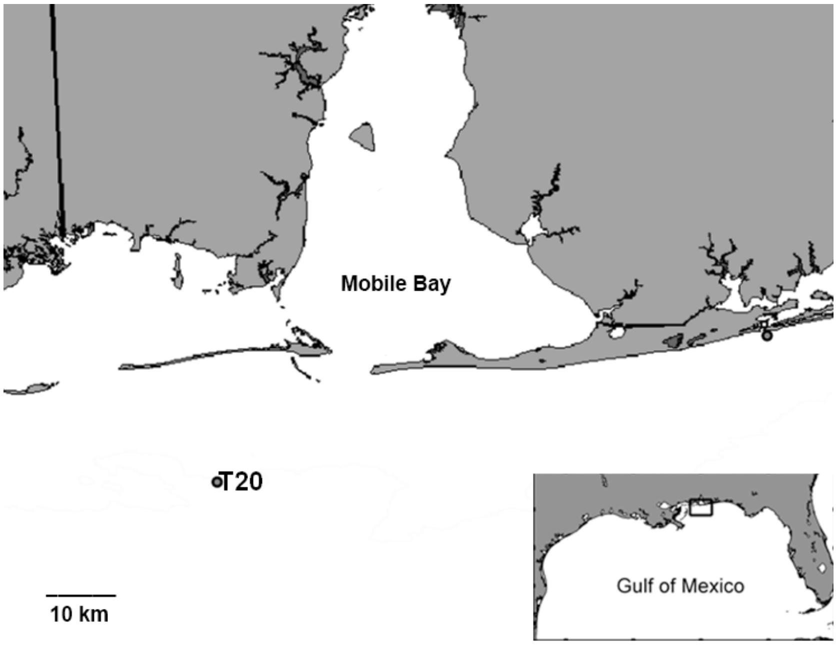

Figure S9. Zooplankton sampling site (T20) off Dauphin Island, Alabama.

Table S1. Northern GoM rivers used to estimate the "Other Rivers" discharge anomaly. River discharge data were retrieved from the U.S. Geological Survey (https://waterdata.usgs.gov).

Name	State	Name	State
Nueces	Texas	Pascagoula	Mississippi
San Antonio	Texas	Alabama	Alabama
Guadalupe	Texas	Tombiggee	Alabama
Colorado	Texas	Perdido	Alabama-Florida
Brazos	Texas	Escambia	Florida
Trinity	Texas	Blackwater	Florida
Sabine	Texas	Yellow	Florida
Neches	Texas	Shoal	Florida
Calcasieu	Louisiana	Choctawhatchee	Florida
Amite	Louisiana	Apalachicola	Florida
Tangipahoa	Louisiana	Chipola	Florida
Bogge Chitto	Louisiana	Aucilla	Florida
Pearl	Louisiana-Mississippi	Ecofina	Florida
Wolf	Mississippi	Fenoholloway	Florida
